# Dementia and autopsy-verified causes of death in racially-diverse older Brazilians

**DOI:** 10.1371/journal.pone.0261036

**Published:** 2021-12-15

**Authors:** Jose M. Farfel, Sue E. Leurgans, Ana W. Capuano, Maria Carolina de Moraes Sampaio, Robert S. Wilson, Julie A. Schneider, David A. Bennett

**Affiliations:** 1 Rush Alzheimer’s Disease Center, Rush University Medical Center, Chicago, IL, United States of America; 2 Department of Pathology, Rush University Medical Center, Chicago, IL, United States of America; 3 Health Sciences Program, Instituto de Assistência Medica ao Servidor Público do Estado (IAMSPE), São Paulo, Brazil; 4 Department of Neurological Sciences, Rush Medical College, Chicago, IL, United States of America; 5 Department of Psychiatry and Behavioral Sciences, Rush Medical College, Chicago, IL, United States of America; Texas State University, UNITED STATES

## Abstract

**Background:**

While dementia has been associated with specific causes of death, previous studies were relatively small autopsy series or population-based studies lacking autopsy confirmation and were restricted to Non-Latinx Whites. Here, we examine the association of dementia with autopsy-verified causes of death in racially-diverse older Brazilians.

**Methods:**

As part of the Pathology, Alzheimer´s and Related Dementias Study (PARDoS), a community-based study in Brazil, we included 1941 racially-diverse deceased, 65 years or older at death. We conducted a structured interview with legal informants including the Clinical Dementia Rating (CDR) Scale for dementia proximate to death. Causes of death were assessed after full-body autopsy and macroscopic examination of the brain, thoracic and abdominal/pelvic organs. Up to four causes of death were reported for each decedent. Causes of death were classified as circulatory, infectious, cancer and other. Logistic regression was used to determine associations of dementia with cause of death, controlling for age, sex, race, and education.

**Results:**

Dementia was associated with a higher odds of an infectious cause of death (OR = 1.81, 95%CI:1.45–2.25), and with a lower odds of a circulatory disease as cause of death (OR = 0.69, 95%CI:0.54–0.86) and cancer as cause of death (OR = 0.41, 95%CI:0.24–0.71). Dementia was associated with a higher odds of pneumonia (OR = 1.92, 95%CI:1.53–2.40) and pulmonary embolism (OR = 2.31, 95%CI:1.75–3.05) as causes of death and with a lower odds of acute myocardial infarction (OR = 0.42, 95%CI:0.31–0.56) and arterial disease (OR = 0.76, 95%CI:0.61–0.94) as causes of death.

**Conclusion:**

Racially-diverse older Brazilians with dementia had a higher odds of an infectious cause of death and a lower odds of cancer and circulatory disease as causes of death than those without dementia.

## Introduction

Dementia is a chronic disabling condition affecting around 50 million people worldwide, the majority of whom live in low and middle-income countries [1]. Latin America is expected to have a greater increase in the prevalence of dementia than developed regions of the globe [2]. We and others have shown that dementia is associated with a higher mortality risk [3–6], and post-mortem studies have reported circulatory, neurologic and infectious diseases, mainly pneumonia, as the most common causes of death in persons with dementia [7–18].

Many groups previously examined differences in causes of death among demented and non-demented subjects and found that subjects with dementia were more likely to die from pneumonia and less likely to die from circulatory disease and cancer [7–18]. However, many of these studies were small autopsy series restricted to a few hundred cases with highly selected participants from tertiary care clinics [7–14]. Others were large population-based studies but lacked autopsy confirmation of the causes of death relying instead on death certificates [15–18]. Most of these studies were restricted to White Europeans living in developed countries. A recent cause-of-death national report in the US based on death certificated shows that the cause-of-death differs by race. While heart disease was the most common cause of death among non-Latinx Whites and Blacks, cancer was the most common cause of death among Latinx [19]. We are not aware of any previous study examining differences of causes of death between demented and non-demented subjects in a diverse population or in Latin America. Here, we use data from a large community-based, post-mortem study to examine the association of dementia with autopsy-confirmed causes of death in a racially-diverse sample of more than 2,000 older Brazilians.

## Materials and methods

### Decedents

Decedents were included from the Pathology, Alzheimer´s and Related Dementia Study (PARDoS). PARDoS enrolls decedents, 18 years or older, who died from non-forensic causes in the State of Sao Paulo. PARDoS is composed of two cross-sectional, community-based studies, with similar eligibility criteria and similar clinical data collection by the same staff facilitating efficient merging of the data, including the study having the same PARDoS name which started in 2020 at the Instituto de Assistencia Medica ao Servidor Publico Estadual (IAMSPE), and the study entitled “Study of Ancestry, Neurodegenerative Diseases and Stroke (SANDS)” which started in 2016 in another institution and was relocated to IAMSPE in 2020 after the former institution terminated its participation in the project in 2019. Both studies were approved by local ethical committees and by Comissão Nacional de Ética em Pesquisa (CONEP), the Brazilian federal ethics committee. Because the index cases were decedents, the study was determined to be of non-human subjects in the United States and IRB exempt in the USA. As previously described [20], enrollment for PARDoS takes place in the State of Sao Paulo and prioritizes Black/Mixed decedents who are 65 years or older at death, and White decedents with fewer than 9 years of education enriched with admixed ancestry. Many decedents, however, were born in different States. The States Paraná, Santa Catarina, and Rio Grande do Sul, located in the southern states of Brazil, are usually less admixed and for this reason, were given a lower priority. This prioritization was implemented by our nurse interviewers who did a screening of the demographics and place of birth before approaching the families.

In Brazil, the informants were considered human subjects of interest. Thus, nurses from the staff identified and approached legal representatives of the decedents to obtain consent while they were waiting for funeral arrangements. PARDoS consent rate was 39.7% of the representatives approached. Representatives who were not able to understand the consent or who were overly distraught were not included. A signed consent, a death certificate filled with autopsy-verified causes of death, and a valid cognitive assessment obtained with an informant were available for 1941 decedents aged 65 years or older at the time of the analyses. The mean age at death of decedents was 79.9 years (SD = 8.9 years; range: 65–106 years) and the mean educational attainment was 4.9 years (SD = 3.8 years; range: 0–25 years); 46.7% of the decedents were women and 31.2% were informant-declared Black (10.0%) or Mixed (21.2%).

### Clinical interview

The informant was asked by a nurse interviewer to participate in an 60-90-minute interview immediately following consent. The clinical interview collects information about the deceased including demographics, and a cognitive assessment using validated questionnaires as detailed below. More than one informant was allowed to participate in the interview; however, the interviewer prioritized answers provided by the informant whose relationship with the decedent was closest and had the most contact with the decedent.

The date of birth and sex were obtained from the decedent’s identification documents; age at death was calculated. Informant-report race was classified as Black, Mixed, or White, according to the Brazilian census criteria [21]. We combined the Black and Mixed groups into one group referred to as Black in this study, following the criteria used by previous authors, for which Mixed race is not a formal census choice. The informant also reported education as the number of years the decedent attended school.

The relationship of the informant to the decedent was recorded, as well as the average number of days a week that the informant had contact with the decedent over the year prior to death and the estimated number of years the informant knew the decedent. Most of the informants were children (75.5%), followed by grandchildren (9.2%), siblings (4.5%), spouses (3.0%), and other (7.8%). Informants knew the decedents for an average of 47.0 years (SD: 12.3) and had contact with the decedents for an average of 2.4 (SD: 3.9) days per week.

### Assessment of dementia and cognitive impairment

The Clinical Dementia Rating (CDR) Scale was used for the diagnosis of dementia proximate to death [22]. We used the informant-specific sections of the CDR structured questionnaire for each of the six cognitive domains of the scale (i.e., memory, orientation, judgment, and problem solving, community affairs, home and hobbies, personal care). Each of the domains was scored on a 5‐point scale as follows: 0, no impairment; 0.5, questionable impairment; 1, mild impairment; 2, moderate impairment; and 3, severe impairment (personal care was scored on a 4‐point scale combining 0 and 0.5 ratings). An algorithm combined the level of the domains affected and resulted in an overall score. A diagnosis of dementia required a CDR > 0.5.

### Autopsy-based causes of death

Brazilian law mandates a full-body autopsy examination for non-forensic cases for persons who cannot obtain a death certificate from a physician. The autopsy procedures include removal, sectioning and macroscopic inspection of the brain, and all thoracic, including lungs and heart and abdominal/pelvic organs, including liver, gallbladder, spleen, pancreas, kidneys, intestines, bladder and sexual organs. The brain and multiple sections were also photographed by the study staff and the images were made available to the medical examiner. Only macroscopic examination was used to determine the causes of death. Microscopic examination was not used for confirming macroscopic findings. The causes of death were determined during the autopsy by the medical examiner who record them in a death certificate required for starting funeral procedures. The examiner was blinded to the clinical interview performed by our staff with informants and had access to very limited medical records which were sometimes used to support secondary causes of death but not the primary cause. The death certificate allows up to four causes of death, starting with the basic or primary cause of death which triggers the sequence of other causes. The causes of death are hand-written by the medical examiner in open fields. Each cause of death was entered in our database and converted to its corresponding International Classification of Diseases (ICD-10) code. While the medical examiners did not follow strict rules, the coding of ICD-10 was done in a structured manner by PARDoS staff. The average number of causes of death reported in our study was 2.02 per case (range 1–4) with 63.6% reporting a second cause, 32.5% reporting a third cause and 11.1% reporting a fourth cause of death. All cases showing signs of trauma were forensic cases and excluded and referred to the forensic medical examiner.

For analysis, we divided causes of death into four major groups: 1. Diseases of the circulatory system (according to the section I. of the ICD-10 (I.00 - I.99)), including heart conditions such as heart failure, hypertensive heart disease, ischemic heart disease; arterial conditions such as regional or systemic atherosclerosis, acute myocardial infarction, acute and chronic strokes; and venous conditions including pulmonary embolism; 2. Infectious diseases included diverse sections of the ICD-10 of infectious origin; 3. Cancer (according to section C. of the ICD-10 (C.00 – C.97)); and 4. Other causes including chronic pulmonary disease such as asthma or chronic obstructive pulmonary disease, chronic kidney failure, cirrhosis, among others.

### Statistical analysis

We first examined the association of dementia with all causes of death by building unadjusted logistic regression models. Three separate models were built using the categories of circulatory disease, infectious disease and cancer as outcomes. We repeated the models for the three groups using only the primary cause of death as a secondary outcome. Next, we built similar models using the specific ICD-10 codes of the five most reported causes of death: 1. heart disease including congestive heart failure, hypertensive and ischemic heart diseases; 2. pneumonia; 3. acute myocardial infarct; 4. arterial disease including significant focal and systemic atherosclerosis; 5. and pulmonary embolism. We repeated the models by adjusting for age at death, sex, education, and race. White race was used as the reference group. Age of death is the most important predictor of dementia [23, 24]. Education is a very established promotor of cognitive reserve, that protects against dementia [25, 26]. Sex and race have also been shown to be related to differential odds of having dementia [27, 28]. We now add unadjusted models into the paper as per the reviewer’s recommendation. These demographic factors may also be associated with specific causes of death. Age is the most important risk factor for cancer incidence and many cancer types [29]. Cardiovascular risk factors and circulatory disease are usually more prevalent in men and African Americans [30]. Lower education is associated with poorer control of cardiovascular risk factors [31]. Finally, to test whether the association of dementia with the groups of cause of death were modified by race we repeated logistic regression models with additional term for the interaction of race and dementia. All analyses considered a nominal threshold of p < 0.05 to determine significance and were conducted using SAS/STAT software, Version 9.4 of the SAS® system for Linux.

## Results

Demographic, clinical characteristics and causes of death are shown in Table 1. Circulatory disease was the most common cause of death when all causes were analyzed, affecting more than three quarters of the sample. Infectious diseases contributed to the cause of death in approximately a quarter of the decedents and cancer in approximately 5%. Circulatory disease was also the most common primary cause of death in two thirds of the cases, followed by infectious diseases in nearly a quarter and cancer in fewer than 5% of the cases. We searched for differences in causes of death by race. We did not find any significant difference of causes of death between Blacks and Whites when considering the major groups of causes and the five more common causes of death verified (all p’s > 0.05).

**Table 1:** Demographic and clinical characteristics of decedents and causes of death.

Characteristic	Total	
	n = 1941	
**Demographics and clinical**		
**Age at death in years, mean (SD)**	79.9	(8.9)
**Women, n (%)**	1035	(53.3)
**Black, n (%)**	606	(31.2)
**Education in years, mean (SD)**	4.9	(3.8)
**Dementia, n (%)**	611	(31.5)
**All causes of death**		
**Circulatory disease, n (%)**	1483	(76.4)
** Heart disease, n (%)**	838	(43.2)
** Atherosclerosis, n (%)**	651	(33.5)
** Acute myocardial infarct, n (%)**	361	(18.6)
** Pulmonary embolism, n (%)**	261	(13.5)
**Infectious disease, n (%)**	540	(27.8)
** Pneumonia, n (%)**	473	(24.4)
**Cancer, n (%)**	114	(5.9)
**Primary causes of death**		
**Circulatory disease, n (%)**	1320	(68.0)
** Heart disease, n (%)**	714	(36.8)
** Atherosclerosis, n (%)**	7	(0.4)
** Acute myocardial infarct, n (%)**	293	(15.1)
** Pulmonary embolism, n (%)**	217	(11.2)
**Infectious disease, n (%)**	360	(18.6)
** Pneumonia, n (%)**	440	(22.7)
**Cancer, n (%)**	46	(2.4)

### Association of dementia with cause of death categories

Table 2 shows the distribution of causes of death in those with and without dementia and the unadjusted logistic regression models examining the association of dementia and causes of death. Circulatory disease was the most frequent cause of death in both demented and non-demented subjects. However, subjects with dementia were less likely to die from circulatory cause of death compared to subjects without dementia. Similar findings were observed when only the primary causes of death were included. Infectious diseases followed circulatory diseases as the second most common group of diseases causing death both in demented and non-demented subjects. In contrast to the findings for circulatory disease, subjects with dementia were more likely to die from infectious diseases when compared to non-demented both when all causes of death were analyzed and when only primary cause was considered. Finally, cancer was the least common cause of death of the three groups of diseases in both demented and non-demented. Similar to the finding for circulatory diseases, demented subjects were less likely to die from cancer compared to non-demented when all causes were considered and when just the primary cause was considered.

**Table 2:** Unadjusted logistic regression models for the association of dementia and causes of death, OR (95%CI), p.

Characteristic	Dementia	Non-Dementia	OR (95%CI)
	n = 611	n = 1330	
**All causes of death**			
**Circulatory disease, n (%)**	443 (72.5)	1040 (78.2)	0.74 (0.59–0.92)
** Heart disease, n (%)**	254 (41.6)	584 (43.9)	0.91 (0.75–1.10)
** Acute myocardial infarct, n (%)**	61 (10.0)	300 (22.6)	0.38 (0.28–0.51)
** Atherosclerosis, n (%)**	179 (29.3)	472 (35.5)	0.75 (0.61–0.93)
** Pulmonary embolism, n (%)**	132 (21.6)	129 (9.7)	2.57 (1.97–3.34)
**Infectious disease, n (%)**	220 (36.0)	320 (24.1)	1.78 (1.44–2.19)
** Pneumonia, n (%)**	203 (33.2)	270 (20.3)	1.95 (1.58–2.42)
**Cancer, n (%)**	17 (2.8)	97 (7.3)	0.36 (0.22–0.61)
**Primary causes of death**			
**Circulatory disease, n (%)**	389 (63.7)	931 (70.0)	0.75 (0.61–0.92)
** Heart disease, n (%)**	217 (35.5)	497 (37.4)	0.92 (0.76–1.13)
** Acute myocardial infarct, n (%)**	43 (7.0)	250 (18.8)	0.33 (0.23–0.46)
** Pulmonary embolism, n (%)**	105 (17.2)	112 (8.4)	2.26 (1.70–3.00)
**Infectious disease, n (%)**	185 (30.3)	255 (19.2)	1.83 (1.47–2.28)
** Pneumonia, n (%)**	165 (27.0)	195 (14.7)	2.15 (1.70–2.72)
**Cancer, n (%)**	5 (0.8)	41 (3.1)	0.26 (0.10–0.66)

Next, we built logistic regression models adjusted for age at death, sex, education, and race to examine the association of dementia with cause of death. We built separate models for each of the three groups of causes of death (circulatory, infectious and cancer). Dementia was associated with a higher odds of an infectious cause of death (OR: 1.81, 95%CI: 1.44–2.25) and lower odds of a circulatory disease (OR: 0.69, 95%CI: 0.54–0.86, p = 0.001) and cancer (OR: 0.41, 95%CI: 0.24–0.71) as causes of death (Table 3). Similar results were found when using only primary cause of death (S1 Table).

**Table 3:** Logistic regression models for the association of dementia and all causes of death adjusted for age at death, sex, education and race, OR (95%CI).

Any cause of death	Circulatory	Infectious	Cancer
**Age at death**	1.00 (0.99–1.02)	1.01 (0.99–1.02)	0.98 (0.96–1.01)
**Male sex**	0.61 (0.49–0.76)	1.37 (1.11–1.69)	1.41 (0.95–2.09)
**Education**	1.04 (1.01–1.07)	0.99 (0.97–1.02)	0.98 (0.93–1.04)
**Race** ^**a**^	0.92 (0.73–1.17)	1.10 (0.88–1.37)	1.33 (0.89 -,1.99)
**Dementia**	0.69 (0.54–0.86)	1.81 (1.45–2.25)	0.41 (0.24–0.71)

^a^White race was used as the reference group.

We also examined whether race modified the association of dementia with cause of death by adding an interaction term of race and dementia in the models. The interaction of race and dementia was not significant in any of the models (all p’s > 0.05)

### Association of dementia with the top five causes of death

Among the five most common specific diseases reported as causes of death, subjects with dementia were more likely to die from pneumonia and pulmonary embolism and less likely to die from acute myocardial infarct and atherosclerosis when compared to non-demented subjects. There was no difference for likelihood of having a heart disease as cause of death between demented and non-demented (Table 2). The results were similar when the primary cause of death was examined. The association of dementia with atherosclerosis as a primary cause of death was not examined as atherosclerosis was a rare primary cause of death.

We also built logistic models to examine the association of dementia with the five most common causes of death adjusted for age at death, sex, race and education. Dementia more than doubled the odds of pneumonia (OR: 2.10, 95%CI: 1.64–2.68) or pulmonary embolism (OR: 2.31, 95%CI: 1.75–3.05) as causes of death and was associated with a lower odds of myocardial infarct (OR: 0.42, 95%CI: 0.31–0.56) or atherosclerosis (OR: 0.76, 95%CI: 0.61–0.94) as causes of death. We did not find any association of dementia with heart disease (OR: 0.89, 95%CI: 0.73–1.09).

Finally, we examined whether race modified the association of dementia with the five most common causes of death. The interaction of race and dementia was not significant in any of the models (all p’s > 0.05).

## Discussion

In this study, nearly 2,000 older decedents enrolled in a community-based study on aging and dementia in the state of Sao Paulo, Brazil, underwent full-body autopsy to determine the causes of their death and their informants were interviewed to provide the diagnosis of dementia using validated scales. We found that dementia is associated with a higher odds of an infectious disease as cause of death, especially pneumonia and with a lower odds of cancer or circulatory disease as causes of death, especially atherosclerosis and acute myocardial infarction.

Previous studies found that the causes of death occurring in subjects with dementia may differ from the causes of death occurring in those without dementia [7–18]. However, most of these studies were either small autopsy-based case-control studies comparing highly-selected demented and non-demented from clinics [7–14], or were large population-based studies in developed countries that lacked autopsy confirmation of the causes of death in all or most of the decedents [15–18]. Further, these studies were performed in developed countries and included a vast majority of White Americans or Europeans. Here, we extend the findings of prior studies in three important ways. First, we increase confidence in our results by confirming the causes of death by a full-body autopsy with macroscopic examination of the brain, and all the organs in the thoracic and abdominal/pelvic cavities. Second, we take advantage of a sample recruited from the community to increase the generalizability of our results. Third, we include a racially-diverse sample with a wide range of educational and socioeconomic backgrounds from a South American Latin country.

Although we found that decedents with dementia had a lower odds of circulatory diseases as causes of death than decedents without dementia, this group of circulatory diseases was by far the leading causes of death both in those with and without dementia. Even in the subjects with dementia, it accounted for nearly two thirds of the deaths as the primary cause. Thus, the importance of circulatory disease to mortality of subjects suffering from dementia cannot be underestimated. Our finding is consistent with other studies showing circulatory diseases as one of the leading causes of mortality in dementia [7–18]. However, we found a higher frequency of circulatory disease as cause of death compared to other studies. The higher frequency of circulatory causes may be related to the distinct profile of our sample with lower education and socioeconomic status which are associated with higher circulatory risk. Further, the autopsy examiner had limited access to minimal clinical data and the microscopic examination was not used for confirming the cause of death [32]. When the group of circulatory diseases was dissected into more specific diagnosis, dementia had a different effect on different circulatory diseases. While dementia was associated with a higher likelihood of venous diseases like pulmonary embolism as cause of death, it was associated with a lower likelihood of arterial diseases such as atherosclerosis and acute myocardial infarction as causes of death. While the lower mobility resulting from dementia may be associated with a higher risk of death from venous thrombosis and pulmonary embolism [33]. The factors associated with a lower mortality due to arterial diseases in demented subjects deserves future investigation with more detailed information on the severity, region and type of arterial disease.

The finding of an association of dementia with a higher odds of an infectious disease as cause of death, especially of pneumonia is supported by prior literature [34]. Pneumonia is particularly more common in advanced stages of dementia when dysphagia is present increasing the risk of aspiration pneumonia [35, 36]. Our finding that dementia is associated with a lower odds of cancer as cause of death is also consistent with the findings from other studies [16–18]. Epidemiologic data from our group and others have suggested an inverse association between dementia and cancer [37–40]. Our study only examined macroscopic cancers and only reported cancers thought to be the cause of death. This in no way reflects the number of people who die with cancer. The same is true for all of the causes of death. Further, the frequency of cancer in our sample may be lower compared to other studies as many subjects dying from cancer already had the cause of death clinically confirmed by their physicians and autopsy was not required in these cases according to Brazilian law. Microscopic tumors or those that were not advanced enough to contribute to death were not reported. However, cancer was the cause of death in more than 5% of the decedents in our study and the organs where most common tumors occur were macroscopically examined. Thus, our findings lend further support to evidence of an inverse relationship between dementia and cancer.

A major strength of this study is the use full-body autopsy to identify causes of death in a large community-based and racially-diverse sample of older Brazilians. Our findings may help allocate resources to prevent causes of death associated with dementia. As circulatory diseases were the most common causes of death in subjects with dementia, the diagnosis, and treatment of cardiovascular risk factors should not be neglected after the diagnosis of dementia. Further, our findings showing that an infectious cause of death is more common in subjects with dementia reinforce the importance of preventive strategies for these conditions, especially for pneumonia. Vaccination, dysphasia assessment, oral hygiene, diet modifications, review of prescribed medications are among the preventive strategies that may reduce the deaths of subjects with dementia by infectious diseases [41–46]. The study also has important limitations. First, autopsy is the gold-standard method to detect causes of death; however, there are causes that are difficult to identify at autopsy. Such causes include some infectious diseases like urinary infection or sepsis that generates non-specific changes in organs and can be better verified by microbiology, or others conditions requiring toxicology, or hypoxic-ischemic brain injury from cardiorespiratory failure. Second, the causes of death are based only in macroscopic examination of the body and the organs. Microscopic examination could confirm or give additional information on the causes of death. Third, some patients with clinically identified cause of death do not come to the autopsy service and this could limit the generalizability of our findings. Forth, we did not examine the survival time between the diagnosis of dementia and death. Finally, we used an informant-based structured interview for the diagnosis of dementia instead of cognitive testing with living subjects. However, informant-based CDR has been previously reported to be highly correlated with antemortem diagnosis and neuropsychological testing [47–49].

## Supporting information

S1 Table Logistic regression models for the association of dementia and primary causes of death adjusted for age at death, sex, education and race, OR (95%CI). (DOCX)
