# Correction: Dementia and autopsy-verified causes of death in racially-diverse older Brazilians

**DOI:** 10.1371/journal.pone.0341697

**Published:** 2026-01-27

**Authors:** The PLOS One Editors

The initial study site for the work reported in [1] is not clearly identified in the Materials and methods section; however, the *PLOS One* Editors note that the Acknowledgments section identifies the Fundação Faculdade de Medicina (FFM), the Autopsy Service (SVOC) at the University of São Paulo’s (USP), and its Medical School (FMUSP), as the site where the project was conducted until September 5, 2019.

The Materials and methods of [1] states that PARDoS (Pathology, Alzheimer’s and Related Dementia Study) is composed of two cross-sectional, community-based studies, with similar eligibility criteria and similar clinical data collection by the same staff facilitating efficient merging of the data, including the study having the same PARDoS name which started in 2020 at the Instituto de Assistencia Medica ao Servidor Publico Estadual (IAMSPE), and the study entitled “Study of Ancestry, Neurodegenerative Diseases and Stroke (SANDS)” which started in 2016 in another institution and was relocated to IAMSPE in 2020. During post-publication discussions, the corresponding author stated that at the time of submission of [1], SANDS had been relocated to IAMSPE, but that all 1941 autopsies included in [1] were carried out at the Sao Paulo Autopsy Service, University of Sao Paulo (SVO-USP) and no data in [1] originates from IAMSPE.

In addition, the affiliation information for the duration of the study may be incomplete. The corresponding author stated that authors JMF and DAB were affiliated to the University of São Paulo Medical School (FMUSP) during the period of SANDS enrollment and data collection for [1].
