# Supplementary material for: Dementia and autopsy-verified causes of death in racially-diverse older Brazilians
Source: PLoS One. 2021 Dec 15;16(12):e0261036. doi: 10.1371/journal.pone.0261036 (PMC8673625; doi:10.1371/journal.pone.0261036)
Supplement: S1 Table — (DOCX) [file pone.0261036.s001.docx]

**Supplementary Table 1. Logistic regression models for the association of dementia and primary causes of death adjusted for age at death, sex, education and race, OR (95%CI)**

| **Primary cause of death** | **Circulatory** | **Infectious** | **Cancer** |
| --- | --- | --- | --- |
| **Age at death** | 0.99 (0.98 - 1.01) | 1.01 (1.01 - 1.03) | 0.98 (0.95 - 1.02) |
| **Male sex** | 0.63 (0.52 - 0.77) | 1.61 (1.29 - 2.01) | 1.16 (0.63 - 2.12) |
| **Education** | 1.02 (0.99 - 1.05) | 0.99 (0.96 - 1.02) | 0.91 (0.83 - 1.01) |
| **Race^a^** | 0.90 (0.73 - 1.11) | 1.15 (0.91 - 1.46) | 1.05 (0.56 - 1.98) |
| **Dementia** | 0.72 (0.58 - 0.89) | 1.84 (1.46 - 2.31) | 0.27 (0.10 - 0.70) |

^a^White race was used as the reference group.
